# Potential of Seaweeds to Mitigate Production of Greenhouse Gases during Production of Ruminant Proteins

**DOI:** 10.1002/gch2.202200145

**Published:** 2023-04-08

**Authors:** Goldy De Bhowmick, Maria Hayes

**Affiliations:** ^1^ Food BioSciences Department Teagasc Food Research Centre Ashtown Dublin 15 D15 KN3K Ireland

**Keywords:** beef, dairy, emissions, polyphenols, seaweed

## Abstract

The potential of seaweed to mitigate methane is real and studies with red seaweeds have found reductions in methane produced from ruminants fed red seaweeds in the region of 60–90% where the active compound responsible for this is bromoform. Other studies with brown and green seaweeds have observed reductions in methane production of between 20 and 45% in vitro and 10% in vivo. Benefits of feeding seaweeds to ruminants are seaweed specific and animal species‐dependent. In some instances, positive effects on milk production and performance are observed where selected seaweeds are fed to ruminants while other studies note reductions in performance traits. A balance between reducing methane and maintaining animal health and food quality is necessary. Seaweeds are a source of essential amino acids and minerals however, and offer huge potential for use as feeds for animal health maintenance once formulations and doses are correctly prepared and administered. A negative aspect of seaweed use for animal feed currently is the cost associated with wild harvest and indeed aquaculture production and improvements must be made here if seaweed ingredients are to be used as a solution to control methane production from ruminants for continued production of animal/ruminant sourced proteins in the future. This review collates information concerning different seaweeds and how they and their constituents can reduce methane from ruminants and ensure sustainable production of ruminant proteins in an environmentally beneficial manner.

## Graphical abstract

This review collates information concerning different seaweeds and how they and their constituents can reduce methane from ruminants and ensure sustainable production of ruminant proteins in an environmentally beneficial manner. Phlorotannins offer potential for use as feed additives to reduce methane emissions from ruminants.

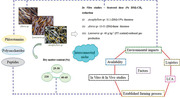

## 1 Introduction

The escalating world population has compelled the food sector to increase productivity and supply in order to meet global demand for food. Ruminants include cattle, sheep, goats, and buffalo and play a very important role in global food security as well as employment for farmers, processors, scientists, and others. The nutritional quality of meat and dairy products is second to none in terms of methods including protein digestibility values calculated using Food and Agricultural Organisation approved methods protein digestibility amino acid score (PDCAAS) or the digestible indispensable amino acid score (DIAAS) method. Indeed, the growing global population has resulted in increased dairy and meat product consumption, and increased livestock farming but also, unfortunately, increased GHG production in the form of methane (CH_4_). Livestock farming produces around 3.1 gigatonnes of CO_2_ equivalents of CH_4_ per annum alone, accounting for up to a third of the total anthropogenic CH_4_ emissions.^[^ ^1^ ^]^ A single cow can generate up to 3 tonnes of CO_2_ equivalents of CH_4_ per year.^[^ ^62^ ^]^ According to the Intergovernmental Panel on Climate Change (IPCC), Fifth Assessment Report (AR5) CH_4_ is considered 28 times more harmful a greenhouse gas (GHG) than CO_2_.^[^ ^80^ ^]^ However, farming also feeds the world, supplying high quality proteins, essential fatty acids, and essential nutrients to consumers. More sustainable livestock farming practices are required to reduce GHG production and global warming and to ensure sustainability of farms.

Seaweeds have potential to contribute positively to prevention of GHG production from ruminants. Previously, researchers including Kinley et al.^[^ ^97^ ^]^ indicated that feed additives could disrupt anaerobic fermentation of feed organic matter in a reduction pathway mainly driven by the methanogenic microbial consortium in ruminants. Altering what ruminants eat (the feed system and ingredients) may significantly impact enteric CH_4_ formation. Methane production from ruminants can be impacted by the antimicrobial, antioxidant activity, and prebiotic potential of feed ingredients. However, in livestock production, the diversity of feed systems and ingredients is dependent on feedstock availability in particular regions, which can vary in terms of climate, pasture types, and indeed associated feed regulatory guidelines.^[^ ^98^ ^]^ In relation to ingredient use to reduce CH_4_ emissions, Patra^[^ ^129^ ^]^ reported that supplementation with ingredients including ionophores, legumes, essential fats, oils, probiotics, tannins, saponins, and other chemical compounds as well as various plant metabolites may help in managing enteric CH_4_ emissions. Anti‐methanogenic activity, however, must be balanced against other dietary considerations such as a decrease in rumen fermentation efficiency, which can result in decreased feed intake that may indirectly affect animal productivity and health.^[^ ^97^ ^]^

Seaweeds are, in several parts of the world, already used as feed additives or livestock feed due to their polysaccharide, mineral, specific vitamin, and bioactive compound content including polyphenols, halocarbons, peptides, and phlorotannins.^[^ ^1^, ^74^ ^]^ The seaweed genus *Asparagopsis* emerged as a potent CH_4_ inhibitory candidate due to the level of bromoform contained in it. Bromoform rich *Asparagopsis* sp. is capable of reducing CH_4_ emissions by up to 98% at concentrations of 0.5 – 2% of dry matter intake inclusion in feeds.^[^ ^1^, ^98^, ^109^ ^]^ Vucko et al.^[^ ^165^ ^]^ reported that the percentage concentration of bromoform is crucial in determining the effectiveness of *Asparagopsis* sp. and should remain above 1 mg g^−1^ of organic matter intake in order to reduce CH_4_ emissions by >99%. Bromoform is, however, perceived to have carcinogenicity and a negative impact on the ozone layer.^[^ ^1^, ^92^ ^]^ Bromoform is released from specialized seaweed gland cells and has anti‐methanogenic activity. It inhibits the cobamid‐dependent methyl‐transfer reactions that cause methane formation. The antimicrobial activity also affects the fermentation profile of the rumen microbiota. This may have a negative health effect including deterioration of the rumen mucosa and transfer of bromoform to meat tissue, milk, and blood (Munoz‐Tamayo et al., 2021).^[^ ^179^ ^]^ *Asparagopsis taxiformis* and *Asparagopsis aramata* are harvested in regions like the Azores and cultivated indoors by companies in Sweden for example today. *Asparagopsis taxiformis* is found mainly in tropical and subtropical locations and is native to Australia. *Asparagopsis aramata* is found in warm temperature regions of the ocean. The genus *Asparagopsis* is highly invasive and *A. armata has* successfully spread in the Mediterranean seas.^[^ ^1^, ^92^ ^]^ In addition, information on the duration of CH_4_ inhibition by *Asparagopsis*‐derived bromoform is not known presently.^[^ ^1^ ^]^ Despite this, in July 2020 New Zealand based dairy processors Fronterra partnered with an algal supply company Sea Forest in a trial to use *Asparagopsis* sp. as a supplement feed for herds in Tasmania during the milk season (https://www.fonterra.com/sea/bh/our-stories/articles/reducing-emissions-with-the-help-of-seaweed.html). Additionally, in March 2022, the California Department of Food and Agriculture (CDFA) Livestock Drug Program issued a letter of no objection to Blue Ocean Barns use of *Asparagopsis taxiformis* for sale as a digestive aid product where Brominata D and O are used as a livestock drug for cattle. The levels of bromoform and iodine in this product are in accordance with what is acceptable by the US Environmental Protection Agency^[^ ^160^ ^]^ tolerance for drinking water but the product cannot be sold as a feed ingredient in California and was not evaluated by CDFA for reduced methane emissions. The product was only reviewed for safety as a livestock drug and not GRAS as a feed additive. Commercial sale and supply of *Asparagopsis* species to cattle producers began in 2022 via CH4Global.

Other seaweeds contain CH_4_ reducing potential due to alternative bioactives to bromoform such as tannins, polyphenols, or prebiotic oligosaccharides. Seaweeds like *Alaria esculenta*, *Ascophyllum nodosum*, and *Chondrus crispus* were reported previously to have a CH_4_‐reducing effect.^[^ ^1^ ^]^ This review looks at the identification and downstream processing of bioactive compounds from various seaweeds and their potential for use in inhibiting CH_4_ production in the production of ruminant protein.

## 2 Seaweed Overview

In general, consumption of seaweeds for human use does not compete with consumption of seaweed species for livestock use. The most commonly consumed seaweeds for human use include Wakame (*Undaria pinnatifida*), Kombu (edible kelp usually species of *Laminaria* like *Laminaria hyperborea* and *Laminaria digitata*), Nori (*P. yezonesis* and *P. tenera*), Dulse (*Palmaria palmata*), Hijiki (*Hizikia fusiforme*), Irish moss (*Chondrus crispus*) and Sea lettuce (*Ulva lactuca*). Species used in animal feeds for their potential to impact methane emissions from ruminants include *Asparagopsis* species including *A. aramata* and *A. taxiformis*, *Laminaria* species, including *Laminaria hyperborea* and *Ascophyllum nodosum*. Potentially, there could be competition for seaweeds for use in both human and animal feed consumption. The species *Asparagopsis taxiformis* is consumed as a delicacy in Hawaii for example. *Ascophyllum nodosum* is primarily used in animal feed and fertilizers currently but is also used for extraction of alginic acid used in the food and biotechnology industry. *Laminaria hyperborea* is also consumed largely in Asia as a flavor ingredient and food additive. Seaweed farming at a mass scale only started during the last decade.^[^ ^14^, ^154^ ^]^ Red seaweeds are a pivotal part of marine ecosystems as they not only represent more than 6000 described species of the marine macrophyte group but also represent various groups of fresh water organisms.^[^ ^42^ ^]^ Most of the marine red algal species are found in the intertidal and subtidal ocean depths up to 40 m or sometimes occasionally 250 m.^[^ ^58^ ^]^ Dominance of the pigments phycoerythrin and phycothcyanin in red algae mask the expression of other pigments, thus providing a unique red color to the plant.^[^ ^58^ ^]^ Species including *Pyropia* sp. *Palmaria* sp. and *Porphyra* sp. are widely used for human consumption and their estimated market value is ≈US$1300 million y^−1^.^[^ ^28^, ^42^ ^]^ Market value of red seaweed alone was estimated to exceed by US$25 billion in 2019.^[^ ^137^ ^]^ Unlike the million dollar polysaccharide industry from the red seaweed, downstream processing of the biomass for some specific platform chemicals that are already discovered are found to be expensive and economically unviable for further mass scale production.^[^ ^45^ ^]^

*Asparagopsis* sp. started grabbing global attention due to their demonstrated bio‐filter efficacy and recent innovation concerning CH_4_ emission reduction when fed to ruminant livestock.^[^ ^92^, ^98^ ^]^ The compound found responsible for such activity was bromoform, a halogenated compound that interfered with the methanogenesis process and was able to effectively, inhibit enteric CH_4_ emissions from livestock.^[^ ^98^ ^]^ Inhibition of methanogenesis by bromoform occurs by blocking the action of key enzymes in the Wolfe cycle—the cycle that describes the stepwise reduction of CO_2_ to CH_4_ in the general reaction CO_2_ + 4H_2_ → CH_4_ + 2H_2_O by rumen hydrogenotrophic methanogenic archaea. Two steps in the Wolfe cycle are catalyzed by coenzyme M methyltransferase (with a cobalamin prosthetic group) and methyl coenzyme M reductase (with nickel tetrapyrrole as a prosthetic group; syn. cofactor F_430_), respectively, and are susceptible to competitive and/or oxidative inhibition. Bromoform reacts competitively with the substrates of coenzyme M transferase and methyl coenzyme M reductase, inhibiting methyl transfer from methyl‐H4MPT to CoM‐SH and the reductive release of methane from methyl‐coenzyme M (Glasson et al., 2022).^[^ ^180^ ^]^ Several in vitro and in vivo studies indicated that *Asparagopsis taxiformis* and *Asparagopsis aramata* are potential candidates for CH_4_ gas emission mitigation,^[^ ^98^ ^]^ however, recent studies also indicated that bromoform has adverse effects on the environment and human health thereby limiting its wide spread use.^[^ ^92^ ^]^ Previous studies have shown that bromoform when released into the atmosphere have a shorter lifetime of 6 months and are referred to as very short‐lived substances (VSLS). These halogenated VSLSs have the catalytic power of destroying the ozone layer into the troposphere and stratosphere. Recently, Muizelaar et al.^[^ ^123^ ^]^ reported the presence of bromoform residues in the milk of lactating cows for the first 9 days with abnormalities observed in the rumen wall upon undergoing histological examinations. The study also suggested that upon feeding 1.26 mg kg^−1^ dry matter of bromoform, residues can be excreted in the urine and milk of lactating cows.^[^ ^123^ ^]^ Although mitigation of enteric CH_4_ seems to be a very promising approach to reduce emissions, further refinement is needed especially considering the associated negative environmental and health implications. Bromoform is also volatile and was suggested by Keng in 2020 and a report by the Danish Government to negatively impact the ozone layer due to its volatility. However, recently a paper by Jia and colleagues^[^ ^88^ ^]^ who focussed on the impact of CHBr_3_ on the stratospheric ozone layer found that emissions from proposed *Asparagopsis* cultivation in Australia and the intensity and impact of CHBr_3_ emissions vary, depending on location and cultivation scenarios. Of the proposed locations, tropical farms near the Darwin region are associated with the largest CHBr_3_ Ozone layer depleting (ODP) values. However, farming of *Asparagopsis* using either ocean or terrestrial cultivation systems at any of the proposed locations in Australia does not have the potential to significantly impact the ozone layer.

Brown seaweeds belong to the Phaeophyceae class and are considered as large assemblage of organisms including both photosynthetic members containing plastids such as diatoms and non‐photosynthetic groups such as slime nets and water molds.^[^ ^4^ ^]^ Brown seaweeds play a very important part in preserving coastal ecosystem just like the rainforests providing habitats and food sources.^[^ ^147^ ^]^ Dominance of the xanthophyll pigment “fucoxanthin” is responsible for the brown color of the seaweed masking expression of other pigments such as chlorophyll a and c, beta‐carotene, and other xanthophylls. The cell wall is typically made up of cellulose and alginic acid (long chain heteropolysaccharide), multicellular organism mostly branched with filamentous thallus.^[^ ^4^ ^]^ The mineral fraction of brown seaweed constitutes up to 36% of its dry matter but brown seaweeds contain little protein when compared to red or green seaweed varieties (on average 5–15% of dry weight).^[^ ^100^ ^]^ Brown seaweeds also biosynthesize fucoidan, fucose‐containing sulfated polysaccharides exhibiting a range of biological activities such as anti‐thrombiotic, anti‐coagulant, anticancer, anti‐viral, anti‐proliferative and anti‐inflammatory.^[^ ^147^ ^]^ A study on crude fucoidan extract from *Sargassum* sp. on inducing apoptosis in Lewis lung carcinoma cells and melanoma B16 cancer cells was affirmed.^[^ ^7^ ^]^ Additionally, it also contains several other bioactive compounds such as omega‐3 poly unsaturated fatty acids (PUFA's, omega‐6 arachidonic acid, and fucosterol.^[^ ^119^ ^]^ Consequently, there is a high chance of lipid oxidation due to the presence of high levels of eicosapentaenoic acid (EPA; 20:5n‐3) and stearidonic acid (18:4n‐3).^[^ ^119^ ^]^

Brown seaweeds are reported to contain polyphenols especially phlorotannins (PTs).^[^ ^119^ ^]^ In a study, the application of 500 µg mL^−1^ of PTs from *Ascophyllum nodosum* resulted in in vitro inhibition of ruminal fermentation of mixed forage or barley grain in a dose‐dependent strategy, wherein reduction in gas production reflected reduced digestion of neutral detergent fiber present in the forage and starch grain diet.^[^ ^169^ ^]^ Greater reduction of gas was observed for neutral detergent‐fiber than for starch grain.^[^ ^169^ ^]^ The study suggested that PT inhibited the ruminal bacteria involved in the fiber digestion to a greater extent than those involved in starch digestion whilst protecting dietary proteins from microbial degradation.^[^ ^169^ ^]^ The probable reason for lowered CH_4_ gas release was due to PT‐protein complex formation reducing deamination of amino acids thereby linearly reducing ammonia‐N accumulation with increasing PT concentration.^[^ ^169^ ^]^ It was observed that PTs were more effective at inhibiting cellulolytic rather than amylolytic bacteria.^[^ ^169^ ^]^ M In in vitro systems, ammonia‐N concentration is the balance between ammonia‐N production, primarily from amino acid deamination, and its utilization for amino acid synthesis by ruminal microbes. In the present experiment,^[^ ^169^ ^]^ the PT‐mediated reduction in ammonia‐N concentration likely resulted from inhibition of the digestive activity of rumen microbes by PT, as well as a direct protective effect of PT on dietary protein. Wong and Cheung also reported that PT in green alga (Alva lactuca) reduced proteolysis of seaweed protein by several digestive enzymes. Accordingly, accumulation of ammonia‐N derived from casein showed maximal values within 4 h of incubation clearly indicating that PT reduced proteolysis of casein and deamination of amino acids throughout the entire incubation period. The casein added to each vial provided a similar amount of N as the diet, but the contribution of casein to ammonia‐N was greater than that from other N sources in the diet during the first 12 h of incubation, indicating that hydrolysis of casein and deamination of its amino acids was more rapid than degradation of other dietary protein. However, the greater reduction by PT of ammonia‐N accumulation from plant N than from casein N suggests a greater affinity of PT for plant protein than for casein. More research is needed to understand in vivo effects of PTs on ruminal fermentation. In another study, it was reported that feeding cows with brown seaweed extracts resulted in other health benefits including a reduction in oxidative stress, stress markers, and ketosis.^[^ ^163^ ^]^

Green seaweeds belong to the Chlorophyceae class and are considered the most diverse group of seaweeds with more than 7000 species. The pigments chlorophyll a and b impart the green color to the organisms but a balance between chlorophyll and other pigments such as β‐carotene and xanthophyll gives a distinct shade to the alga. Green seaweeds contain less varieties of secondary metabolites – less than 300 compounds compared to the red and brown seaweeds.^[^ ^1^ ^]^ Green seaweeds are mostly found in the light abundant areas such as shallow waters and pools with widely fluctuating temperatures, sea conditions, irradiance, and salinity.^[^ ^95^ ^]^ *Ulva* sp., *Codium* sp., *Chaetomorpha* sp., and *Cladophora* sp. are the main seaweed genus found globally and among them, *Ulva* sp. is the most common genera found in brackish waters.^[^ ^122^ ^]^ *Ulva* sp. abundantly grows well in nutrient rich areas mainly in shallow waters causing dangerous algal blooms blocking watercourses, destroying marine eco‐system, and releasing poisonous vapors.^[^ ^122^ ^]^ In spite of such adverse effects, commercial cultivation of *Ulva* sp. is still favored due to its high quality protein and rich soluble ulvans‐sulfated polysaccharides located in the cell wall of the alga. Ulvans are commercially very important as they have multifunctional properties including anticoagulation, antiviral, anti‐inflammatory, antioxidant, antibacterial, antihyperlipidemic, immunomodulatory, and anticancer activities.^[^ ^1^, ^122^ ^]^ Abbott et al.^[^ ^1^ ^]^ reported that functional carbohydrates are useful for microbial production allowing production of a wide range of chemicals and intermediates such as organic acids, alcohols, and other biomaterials. Additionally, the presence of phenolics (phloroglucinol) and other important pigments (chlorophylls and carotenoids) in substantial quantities makes them a perfect candidate for scavenging free‐radicals.^[^ ^122^ ^]^ It has also been reported that some *Ulva* sp. were used as livestock feeds previously and upon addition to their diets abdominal and subcutaneous fat was reduced whilst meat quality in chickens fed *Ulva* sp. was improved.^[^ ^178^ ^]^ Green seaweeds are also considered an excellent tool for bioremediation and water treatment due to their high nutrient and metal absorption capacities.^[^ ^178^ ^]^ They also tend to contain bioactive compounds having biostimulant properties and are considered safe for agricultural applications.^[^ ^178^ ^]^

## 3 Bioactive Compounds from Seaweeds

Enteric CH_4_ formation is a highly complex process wherein ruminant bacteria, archaea, protozoa, bacteriophage, and fungi ferment simple and complex plant carbohydrates to volatile fatty acids (VFAs) and hydrogen (H_2_). The methanogenic bacteria also known as methanogens convert H_2_ into CH_4_, a metabolic end‐product. Numerous approaches focusing on reducing enteric CH_4_ were investigated including animal nutrition, vaccine development, and genetics and management, however, among the three approaches improving animal efficiency is considered the most viable approach. In one study concerning feed additive use addition of the compound 3‐nitrooxypropanol (3‐NOP) at a dose of 2 g d^−1^ and 25–125 mg kg^−1^ (feed dry matter) to beef and sheep reduced CH_4_ emissions by 30–40%.^[^ ^143^ ^]^ The inhibitor 3‐NOP inhibits the methanogenesis process by inactivating the enzyme methyl‐coenzyme M reductase used by the archaea. Likewise, bromoform acts as a structural analog of methyl‐CoM inhibiting methyl‐coenzyme M reductase.^[^ ^128^, ^181^ ^]^ Due to the lack of regulatory approvals in most regions, legislative mandate inappropriateness, and effects on animal productivity widespread usage of 3‐NOP was further hindered in the USA.^[^ ^163^ ^]^ Feeding seaweed extracts to livestock gained renewed global interest recently due to the macromolecular chemical complexity and diversity of seaweeds, which, can act as prebiotic promoters, and CH_4_ inhibitors.

## 4 Biogenic Halocarbons and Bromoform

Biogenic halocarbons like diiodomethane, chloroiodomethane, and bromoiodomethane including bromoform are produced through anthropogenic and biogenic processes in some seaweeds. Their role in the seaweed is mainly to act as defense compounds and antioxidants and they have antibacterial and anti‐herbivory functions.^[^ ^92^ ^]^ However, amongst phytoplankton species, seaweeds contribute 70% of the total biogenic halocarbons in the world's atmosphere.^[^ ^36^ ^]^ Keng et al.^[^ ^92^ ^]^ exquisitely summarized studies on stress subjugated halocarbon production in seaweeds. The halocarbons bromoform (CHBr_3_), dibromomethane (CH_2_Br_2_), methyl iodide (CH_3_I), diiodomethane (CH_2_I_2_), bromochloromethane (CH_2_BrCl), bromodichloromethane (CHBrCl_2_), and dibromochloromethane (CHBr_2_Cl) are found in several red, brown and green seaweeds.^[^ ^93^ ^]^ In red seaweeds, 90% of halogenated secondary metabolites are either brominated or chlorinated. These compounds are produced due to ecological stress triggered by epiphytic bacteria.^[^ ^92^ ^]^ Further, it was noticed that seaweeds significantly release CHBr_3_ particularly from the species belonging to the tropical, temperate, and polar‐regions, however, studies also indicate that bromo‐and iodinated halocarbon emissions are correlated to photosynthetic activity.^[^ ^93^ ^]^ Perhaps, Mithoo‐Singh et al.^[^ ^118^ ^]^ rightly pointed out that lower pH had a significant effect on the very short‐lived halocarbon (VLSH) emission and subsequently, in their study, it was observed lower seawater pH levels resulted in higher halocarbon release from *Padina australis* and *Sargassum* sp. Moreover, the effect of halocarbon emission was also found to be a species dependent phenomenon. For example, CHBr_3_ emission from *Eucheuma denticulatum* was much higher under higher irradiance in comparison to the Antarctic seaweed *Enteromorpha compressa* that emitted higher amount of halocarbon under low light conditions.^[^ ^93^ ^]^ Other important environmental factors effecting halocarbon emissions include desiccation, oxidative stress, tissue age, irradiance, tissue wounding/grazing, and photosynthetic activity.^[^ ^118^ ^]^ Overall, it was observed that not only type of seaweed, specific species, and geographical location but also environmental factors had a significant impact on total CHBr_3_ emissions.

### 4.1 Positive Effects of Bromoform and Halocarbons on CH_4_ Emissions

3‐Nitrooxypropanol (3‐NOP), bromoform and seaweed bioactives are ionophores—low molecular weight natural products that make membranes permeable to specific ions. 3‐NOP targets methyl‐coenzyme M reductase, which catalyzes the last step of methanogenesis in methanogens. Bromoform and other methane halogenated analogs react with vitamin B_12_ to block the last step of methanogenesis in methanogens. Other seaweed bioactives like polysaccharides, proteins, peptides, alkaloids, bacteriocins reduce CH_4_ production by antimicrobial action/suppression and antioxidant activities.^[^ ^1^ ^]^

**Figure 1:**
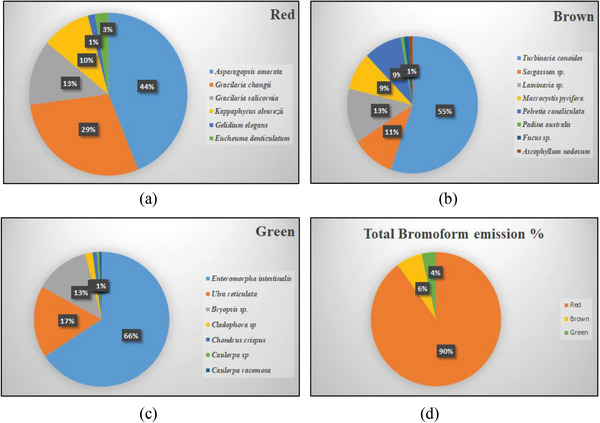
a–c) Percentage distribution of CHBr_3_ concentration (average value) in different species of red, brown, and green seaweed; d) Comparison of CHBr_3_ concentration among the three major classes of seaweed. The value of percentage distribution was calculated on the basis of fresh weight of CHBr_3_ concentration (pmol g^−1^ FW h^−1^) values taken from Keng et al.,^[^ ^92^ ^]^ Thapa et al.,^[^ ^157^ ^]^ and Mithoo‐Singh et al.^[^ ^11^, ^36^, ^38^, ^118^ ^]^

**Figure 2:**
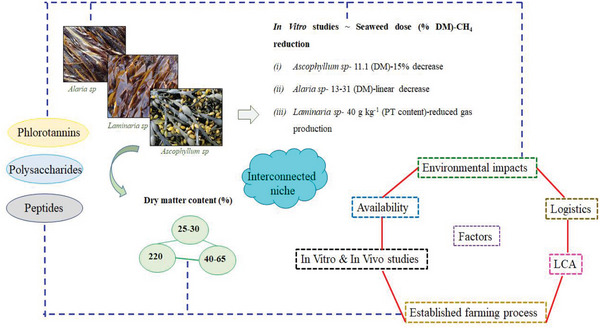
Interconnected niche of CH_4_ mitigation strategies using seaweeds. Seaweeds including the brown species are rich in PTs, complex polysaccharides, and peptides that have demonstrated reduced CH_4_ production from ruminants previously.

Bromoform acts as a structural analog of methyl‐CoM and this cofactor competitively inhibits the Methyl‐CoM reductase (Mcr) enzyme in the methanogenesis pathway carrying the cobamide‐dependent methyl group.^[^ ^128^ ^]^ By inhibiting Mcr activity, reduced CH_4_ production was observed,^[^ ^128^ ^]^ therefore, seaweeds containing bromoform showed significant reduction of enteric CH_4_ emissions. Additionally, the presence of haloperoxidases particularly bromoperoxidase enzyme was comparatively higher in red and brown seaweeds enhancing CHBr_3_ production and release.^[^ ^157^ ^]^ Among the three categories of seaweeds, red seaweeds are known to have the highest CHBr_3_ emission rates (0.71–4960 pmol g^−1^ FW h^−1^) followed by the brown (0.1–1100 pmol g^−1^ FW h^−1^) and the green seaweeds (0.4–344 pmol g^−1^ FW h^−1^).^[^ ^92^ ^]^ **Figure** **1** shows the percentage distribution of CHBr_3_ concentration in each variety of the three major types of seaweeds. The temperate red seaweed *Asparagopsis armata* had a CHBr_3_ emission rate of 4960 pmol g^−1^ FW h^−1^ followed by *Gracilaria*, *Kappaphycus*, and *Eucheuma* (Figure 1a). Several studies indicate that CHBr_3_ can lower enteric CH_4_ emissions in livestock by blocking the methanogenesis process in ruminant microbes. An in vitro analysis suggests that upon inclusion of *Asparagopsis* with 1% dry matter in the diet of dairy cows a 67% reduction in CH_4_ production was observed.^[^ ^144^ ^]^ In another study, when *Asparagopsis taxiformis* was included in the feed of Brahman‐Angus cross steers at a rate of 0–0.2% over a period of 90 days a 40 to 98% reduction in CH_4_ was observed for 0.1 to 0.2% of receiving steers.^[^ ^98^ ^]^ In this study, it was observed that with the increment in *Asparagopsis* inclusion rate from 0%, 380%, and 1700% without compromising dry matter intake enteric H_2_ emission increased steadily, thereby reducing the overall CH_4_ emissions.^[^ ^98^ ^]^ Additionally, total volatile fatty acids were not affected whilst acetate was decreased in favor of propionate resulting in a favorable decrease in acetate: propionate ratio of 14%, 29%, and 35%.^[^ ^98^ ^]^ Also, no negative impact of seaweed usage on meat quality, rumen function, feed conversion efficiency, and feed intake capacity was observed, whilst steady weight gain was demonstrated.^[^ ^98^ ^]^ Similarly, among the other red algae *Gigartina* sp. also reduced enteric CH_4_ emissions (63% less CH_4_) when used with corn silage and meadow hay (44% less CH_4_) as the basal substrate. Brown and green seaweeds also were shown to reduce CH_4_ emissions from ruminants but reductions in CH_4_ were significantly lower than those observed with *Asparagopsis* sp.^[^ ^114^ ^]^ In fact, in some cases, CH_4_ production was further enhanced when seaweeds like *Laminaria ochroleuca*, and *Saccharina latissimi* were incubated with meadow hay instead of corn silage.^[^ ^114^ ^]^ All these studies indicate that seaweed species containing a higher percentage of volatile halogenic compounds were able to effectively reduce enteric CH_4_ emissions, however, in vitro studies also suggested that basal diet is important in determining the interaction of seaweed compounds with the chosen substrate (**Figure** **2**).

### 4.2 Negative Effects of Bromoform and Halocarbons

Thousands of halogenated compounds are found in seaweeds generated by biotic and abiotic stress. These halocarbons play a crucial role in regulating tropospheric and stratospheric ozone, while some of the generated halocarbons bio‐accumulate toxic anthropogenic‐persistent organic pollutants (POPs).^[^ ^24^ ^]^ Very short‐lived halocarbons (VSLH) and bromoform influence the atmospheric chemistry by radiative forcing via cloud nuclei formation and contribute to local weather change.^[^ ^171^ ^]^ Such events can potentially negatively alter ozone abundance and radiative impact accelerating climate change.^[^ ^92^ ^]^ Additionally, the Environmental Protection Agency (EPA, USA) and European Commission (EC) have demonstrated the toxicological impact of chlorinated volatile organic compounds leading to cancer incidences, threatening human health and animal life.^[^ ^77^ ^]^ There are also issues regarding cultivation of some red seaweeds. If *Asparagopsis* sp. known to reduce CH_4_ due to CH_3_Br_3_ content were to be used as a feed ingredient then cultivating *Asparagopsis* itself would be a big challenge. Some, of the problems concerning *Asparagopsis sp*. application as feed ingredients can include i) the level and concentration of bromoform which may negatively impact stratospheric ozone;^[^ ^69^ ^]^ ii) Carcinogenicity;^[^ ^128^ ^]^ iii) bromoform activity loss (volatilization) during the post‐harvest phase; iv) downstream process stability of the compound; and v) complex logistics and associated biomass production costs. Demonstrated carcinogenicity of bromoform in humans and animals was reported previously by Risher et al.^[^ ^141^ ^]^ where chronic exposure to CH_3_Br_3_ lead to intestinal tumors in female rats and liver tumors in female and male mice (143 mg kg^−1^ day^−1^–5 days per week for 2 years). Additionally, Mangusson et al. (2020),^[^ ^182^] reported loss of CH_3_Br_3_ activity through the volatilization process and proposed an effective *Asparagopsis* biomass processing strategy using vegetable oil based storage methods. The study showed that for 120 g of biomass 100 mL of vegetable oil was required (Mangusson et al., 2020), thus if 1000 tonnes of biomass is used then 1000 tonnes of oil would also be required. In reality, the use of *Asparagopsis* sp. would demand additional use of vegetable oil with increased processing costs to preserve CH_3_Br_3_ content in the biomass. It is envisaged that in order to use seaweeds as feed ingredients a minimum of 1% of the Dry Matter Intake (DMI) is required. Aquacultural development of seaweeds is required to ensure a sustainable supply of biomass. Vijn et al.^[^ ^163^ ^]^ calculated the volume of seaweed required for livestock feed at an inclusion rate of 1% of the diet and found that in USA where there are over 93 million cattle, ≈305–339 million metric tonnes DMI per year is required. Consecutively, it was estimated that for 305–339 million metric tonnes DMI per year around 3–3.4 million metric tonnes of dry seaweed per year is required for an inclusion rate of 0.01% of the diet of cattle that is considered.^[^ ^163^ ^]^ Furthermore, it was also reported that ≈31% of ingested iodine from *Asparagopsis* species could be transferred to milk thereby significantly increasing the iodine content far greater than the reported limit.^[^ ^103^ ^]^ Additionally, Lean et al.^[^ ^103^ ^]^ during their meta‐analysis study also pointed out that feeding *Asparagopsis taxiformis* to lactating cows at a rate higher than 0.5% resulted in significant increases in Iodine (five times higher) and bromide (eight times higher) content in milk. Given this, a child less than 3 years old at the consumption rate of 1 L d^−1^ could potentially end up consuming 15 times more iodine and bromide crossing the recommended daily intake limit. Recently, another study confirmed the presence of bromoform in milk and urine upon feeding *Asparagopsis* sp. to twelve lactating Holstein‐Friesian dairy cows, however, bromoform did not accumulate in the animal tissue.^[^ ^123^ ^]^

According to Reisinger and colleagues (Reisinger 2021)^[^ ^183^] methane reduction in cattle using 3‐NOP has limited impact on production. The impact of seaweeds on methane production and animal health is poorly documented although it is thought that seaweeds can have anti‐helmintic and other health benefits. Bromoform is a carcinogen and could have negative impact on animal health and food quality but this is disputed by authors and conflicting views are often presented.

Seaweeds where the actives are alternatives to bromoform (i.e., phlorotannins, lipids) also pose some hazards in terms of use as animal feeds. Iodine levels must be carefully measured and must comply with EU regulations regarding the permitted levels allowable in feeds for ruminants and other animals. The FEEDAP Panel recommends that the maximum iodine contents in complete feed be reduced as follows: dairy cows and minor dairy ruminants, 2 mg I kg^−1^; laying hens, 3 mg I kg^−1^; horses, 3 mg I kg^−1^; dogs, 4 mg I kg^−1^; cats, 5 mg I kg^−1^. The quantity of heavy metals present in feed is also important and Arsenic is particularly important in terms of seaweed use as feed.

## 5 Bacteriocins

Bacteriocins are ribosomally synthesized, naturally occurring peptides produced by lactic acid bacteria (LAB) that are usually heat‐stable and are toxic to closely related bacterial strains.^[^ ^15^ ^]^ Having a wide spectrum of activity, bacteriocins are often considered as specific targeted bactericidal compounds. Bacteriocins can be either exogenous or endogenous. Nisin, an exogenous bacteriocin produced by *Lactococcus lactis* has shown a significant reduction (36% reduction) in ruminant methyl production by stimulating propionate production levels by increasing the propionate to acetate ratio.^[^ ^46^ ^]^ However, the duration of CH_4_ reduction decreased as ruminal bacteria became nisin resistant over time raising concerns regarding its long‐term efficacy in feed applications. An old study by Kalmokoff et al.^[^ ^91^ ^]^ conducted on 50 different strains of *Butyrivibrio* sp. suggested that about half of the bacterial assemblages were able to produce bacteriocins that were able to reduce CH_4_ production. However, the mechanism of action of the bacteriocin on ruminal microbes including archea was not determined and wide spread application for this purpose is currently being reviewed. There is a reason methane is produced in animal rumen, and this is to mitigate the excess of H_2_ produced during carbohydrate fermentation. Eliminating methane‐producing bacteria may impact rumen buffering and microbial protein synthesis (which is extremely important for ruminants). This often affects fiber digestion, intake, etc. In addition, some bacteria may stop synthesizing methane (shifting to another pathway), and new bacteria might grow instead. Nevertheless, many things change in the rumen environment, which should be considered and discussed. Several past so‐called methane solutions did not work because they focused only on methane production without paying much attention to their consequences to the rumen environment.

In seaweeds, bacteriocin producing bacteria are found bound on the seaweed surface. Examples of seaweed derived bacteriocins include the lantibiotic—lichenicidin produced by lactic acid bacteria (LAB) and *Bacillus* sp., associated with brown algae.^[^ ^81^ ^]^ It has also been reported that around 34% of the bacteriocins isolated from seaweed surfaces resulted in antibacterial activity against fouling bacteria present in net‐cages in coastal waters.^[^ ^81^ ^]^ In another study, it was found that the bacteriocin produced by *Staphylococcus haemolyticus* MSM isolated from *Padina tetrastomatica*, *Ulva lactuca*, and *Gracilaria corticata* displayed antimicrobial activity against fish pathogens.^[^ ^156^ ^]^ Presently, the concept of bacteriocin usage to control the microbiota of the rumen is very new and therefore, further detailed investigation must be encouraged.

## 6 Phlorotannins

Brown seaweeds are the only species that are able to produce the polyphenols known as phlorotannins (PT). Phlorotannins consist of phloroglucinol (1‐, 3‐, 5‐trihydroxybenzene) polymerised units produced via the acetate‐malonate pathway.^[^ ^176^ ^]^ The molecular size of PTs varies between 126 Da and 650 kDa, and their concentrations in the epidermal cortex of brown seaweeds vary from 0.5–30% of total dry weight of the cell. Such variations in concentrations could be related to external factors such as habitat, algae size, age, tissue type, and environmental conditions including season, nutrient type, light, salinity, and water depth.^[^ ^37^ ^]^ PTs are categorized into two major categories—condensed and hydrolyzable tannins that play an adaptive role in defense. Further, PTs are classified according to the linkages formed between the phloroglucinol monomeric units into four main groups include fucols (with a phenyl linkage), fuhalols and phlorethols (with ether linkages), fucophloroethols (with ether and phenyl linkges), and eckols (with dibenzodioxin linkages).^[^ ^44^, ^176^ ^]^ PTs have some similarity to terrestrial tannins but structural differences are quite prominent.^[^ ^57^ ^]^ Due to protein binding activity, it was hypothesized that PTs were responsible for reduced digestibility of feeds in ruminants. However, later studies confirmed that inclusion of up to 5% PT extracts from *Ascophyllum nodosum* and *Fucus serratus* in pig feed had no significant effect on digestibility of the feed.^[^ ^57^ ^]^ Indeed, PT inclusion at 2–4% of total feed has shown benefits to rumen nutrition including supporting protein metabolism and reducing bloat as well as anthelmintic activity against gastrointestinal parasites.^[^ ^167^ ^]^ **Table** **1** collates information on the effect of PTs on rumen microbiota. Although PTs showed reduction in growth in cellulolytic ruminal bacteria^[^ ^166^ ^]^ the mechanism of action is not known. It was hypothesized that the mode of action of PTs was related to different bacterial cell wall structures and chemical compositions as the primary site of inhibitory action by tannins is the bacterial cell wall.^[^ ^166^ ^]^ In another study Wang et al.^[^ ^169^ ^]^ reported that feeding lamb and other cattle with dried *Ascophyllum nodosum* containing 50 g PT kg^−1^ dry matter showed no adverse effect on feed intake or growth rate but the minimal inhibitory concentration of in vivo PT was much higher than that observed in vitro study. There is a dearth of information on in vivo PT effect on the ruminal fluids and therefore, more studies needed to be done to draw a direct conclusion regarding its long term effect.

**Table 1:** Collates information on the effect of phlorotannins (PTs) on rumen microbiota

PTs source (algae species)	PTs content/% application	Effect on rumen microbiota	Ref.
*Ascophyllum nodosum* (Brown)	500 µg mL^−1^	i) Reduction in growth of *Fibrobacter succinogenes* by 65–83% ii) Reduction in growth of *Ruminococcus albus* iii) No effect on *R. flavefaciens*	[166]
*Ascophyllum nodosum* (Brown)	2.44 g kg^−1^ (5% inclusion rate ≈1.56 gL^−1^)	Reduction in growth of nitrogen utilizing bacteria	[20]
Stormtoss shoreweed containing *Fucus vesiculosus* (Brown)	42.3 mg kg^−1^	Reduced growth due to deamination of dietary and microbial amino acids	[96]
*Ascophyllum nodosum* (Brown)	50–100 g mL^−1^	Bactericidal effects were observed due to alteration of the bacterial cell wall	[166]
*Ecklonia stolonifera*, *Eisenia bicyclis*, *Sargarssum fulvellum*, *Undaria pinnatifida*, and *Sargassum fusiforme*	5% inclusion rate	Detrimental effect on cellulolytic bacteria	[41]
*Saccharina latissimi* (Brown)	‐	Reduced growth‐PTs present in the alga may have formed complexes with proteins and fibers	[65]
*E. stolonifera*	2% inclusion rate	No detrimental effect on rumen fermentation characteristics and microbial population	[104]
*Laminaria digitata*	40 g kg^−1^	Reduced methane gas production protecting from ruminal fermentation and methanogenesis	[164]
*Ascophyllum nodosum* (Brown)	113 g d^−1^	Inhibited ruminal proteolysis	[11]

## 7 Lipids

Fat content of seaweed varies from 1.6–2.6% of their fresh weight; Brown seaweeds contain around 1.6% lipid, red seaweeds 1.3%, and the green seaweeds 2.6% lipid.^[^ ^1^, ^25^ ^]^ Amongst all the species *Ascophyllum nodosum* is known to have a fat content of 38 g kg^−1^ of its dry matter compared to *C. crispus*, which showed a fat content of 7 g kg^−1^ of its dry matter.^[^ ^1^, ^25^ ^]^ Maia et al.^[^ ^114^ ^]^ evaluated the effects of five seaweeds including *Ulva* sp., *Laminaria ochroleuca*, *Saccharina latissima*, *Gigartina* sp., and *Gracilaria vermiculophylla* on in vitro ruminal fermentation and CH_4_ gas production at an inclusion rate of 25% and 75% of its dry matter. Differences in gas production were observed when supplemented with meadow hay and corn silage along with different types of seaweed. It was also observed that among the lipid molecules particularly EPA and docosahexaenoic acid (DHA, C22:6, n‐3) had inhibitory effects on the rumen methanogenesis process. Documented evidence on the inhibitory effect of microalgal DHA and EPA on the rumen methanogenesis process indicated that supplementing microalgae to animals resulted in incomplete biohydrogenation leading toward accumulation of hydrogenation intermediates that interfered with the ruminal population resulting in lower CH_4_ emissions.^[^ ^17^ ^]^ The basal diet also has an effect on gas production and release as was evident in some studies wherein PUFAs from the brown macroalga *Sargassum* sp. (*S. natans*; *S. fluitans*) solely resulted in inhibition of ruminal methanogenesis.^[^ ^117^ ^]^ Furthermore, Patra et al.^[^ ^130^ ^]^ reported that any by‐product containing high concentration of lipids including C12:0, C18:3, and PUFAs in supplements such as brewer's grains, grape marc, and hominy meal may result in cost‐effective ways to mitigate ruminal CH_4_. However, in their study it was highlighted that along with the fatty acids, grape marc was also rich in tannins and other plant secondary metabolites that jointly supported ruminal microbe inhibition.^[^ ^130^ ^]^ Additionally, all these examples also suggest that brown macroalgae have potential for use in CH_4_ mitigation.

Several studies conducted concerning supplementation of low levels of lipid in animal diets have shown sharp decreases in CH_4_ gas production and increased density of diets and animal productivity.^[^ ^16^, ^30^ ^]^ The hypotheses in these studies were that inhibition of methanogenesis was due to the replacement of rumen fermentable organic matter in the ruminant diet through biohydrogenation of unsaturated fatty acids providing an environment that helps in lowering ruminal methanogens and protozoa content.^[^ ^16^, ^30^ ^]^ Hydrogen (H_2_) produced during biohydrogenation often competes with the methanogenesis process resulting in decreased CH_4_ gas emissions.^[^ ^16^, ^30^ ^]^ Although stoichiometric analysis suggests that 1–2% of this H_2_ is used for the inhibition process, less than 4% of total dry matter intake is required to achieve 20% reduction in CH_4_ emissions. Another study suggested that suppression of unsaturated fatty acids was due to a direct inhibitory effect on methanogenic Archaea such as *Methanobrevibacter smithii*, *Methanobrevibacter ruminantium*, and *Methanosphaera stadtmanae*.^[^ ^30^ ^]^ Lipid supplementation with any kind of plant or animal based oil is a costly method to implement at a large scale and may result in specific drawbacks such as poor fiber digestibility, depressed milk fat synthesis, and altered fatty acid composition of the product. However, all these factors were equally dependent on accessory diet supplementation.^[^ ^30^ ^]^

## 8 Carbohydrates

The most abundant carbohydrate found in seaweeds is cellulose or starch accounting for 60–70% of the total biomass. Algal polysaccharides play an important role in rumen fermentation—they can act as a prebiotic with the ability to enhance antimicrobial activity by stimulating growth of gut beneficial bacteria. *Ascophyllum nodosum* can alter rumen fermentation and drastically affect enteric CH_4_ production.^[^ ^177^ ^]^ In another study, *Ascophyllum nodosum* supplementation in dairy cows resulted in ruminant bacteria proteolytic inhibition.^[^ ^11^ ^]^ Plausible reasons for such inhibition were due to the combined effect of PTs and complex carbohydrates.^[^ ^11^ ^]^ Another important study on specific polysaccharides including the anionic sulfated polysaccharide fucoidan, carboxylated polysaccharide alginate, laminarin, and mannitol found in brown seaweed (*S. fusiforme*) resulted in decreased CH_4_ production by providing fiber and minerals to the ruminant diets.^[^ ^40^ ^]^ This study also revealed that by providing 1–10% inclusion rate of *S. fusiforme*, volatile fatty acid (VFA) concentration increased significantly and that helped in reducing total CH_4_ emissions.^[^ ^40^ ^]^ These studies discussed the possibility of shifting the metabolic pathways of methane producing bacteria but need further analysis. All these examples suggest that seaweed complex carbohydrates could be used to reduce CH_4_ production from ruminants.

Dietary carbohydrate composition influences rumen fermentation patterns and enteric CH_4_ production and releases into the atmosphere. Due to the differences in rumen fermentation pathways starch and sugar also differ in degradable patterns resulting in differentiated VFA which affects the CH_4_ production rate in the rumen.^[^ ^31^ ^]^ Some studies indicated that by replacing maize silage with grass silage CH_4_ production per kg of organic matter increased significantly.^[^ ^32^ ^]^ Perhaps, during this low‐fiber and high‐fiber grass silage comparison study, a lower proportion of propionic acid and a higher proportion of acetic acid were observed which influenced high CH_4_ production.^[^ ^32^ ^]^ Earlier, Boadi et al.^[^ ^29^ ^]^ observed that propionic acid acted as an alternative H_2_ sink thereby reducing the amount of H_2_ transformed into CH_4_ whereas acetic acid enhanced CH_4_ production. Several studies on various carbohydrate sources on rumen fermentation were reported and all these studies indicated that high dietary starch content inevitably increased propionate and helped in lowering ruminant CH_4_ production, whereas dietary sugar always increased butyrate proportions and in turn CH_4_ emissions.^[^ ^32^, ^87^, ^126^ ^]^ Tree foliages such as *Leucaena leucocephala* (River tamarind), *Piscidia piscipula*, *Neomillspaughia emargiata*, and *Tabernaemontana amygdalifolia* when used as supplements showed a reduction in enteric CH_4_ production by 15.6–31.6% whilst increasing the proportion of propionic acid.^[^ ^6^ ^]^ Previous studies also indicated that rumen pH played a pivotal role in the methanogenesis process as it was observed that acidic pH favored propionate fractions helping in reducing hydrogen sink and decreasing CH_4_ production.^[^ ^126^ ^]^ Keeping these hypotheses in mind Børsting et al.^[^ ^31^ ^]^ conducted trials using wheat and molasses in dairy cows and observed that molasses sources at a 25% inclusion rate showed higher CH_4_ production in comparison to wheat sources. Although the exact mechanism of complex or high fiber carbohydrate action on rumen fermentation cannot be elucidated, the study shows that complex carbohydrates with high fiber content seem to reduce enteric CH_4_ production.

## 9 Alkaloids and Saponins

Alkaloids and saponins are plant metabolites with varying biological activities including antimicrobial activity (Belanche et al., 2015).^[^ ^21^ ^]^ Alkaloids are nitrogenous compounds whilst saponins include compounds that are glycosylated steroids, triterpenoids, and alkaloid steroids that are capable of producing foams in aqueous solutions like soaps (Belanche et al., 2015).^[^ ^21^ ^]^

The seaweed *Caulerpa* was described in previous studies and alkaloids that had anti‐inflammatory activity in a murine model of carrageenan‐induced peritonitis were identified.^[^ ^151^ ^]^ Caulerpin, an indolic alkaloid from the genus *Caulerpa* is well studied and is also found in the red seaweed *Chondria armata*.^[^ ^151^ ^]^ Other alkaloids identified in seaweeds include racemosin A, B, and C, and caulersin.^[^ ^151^ ^]^ Additionally, red algae of the genus *Gracilaria* and *Laurencia* emerged as important source of biologically active alkaloid compounds.^[^ ^151^ ^]^ Dictyospiromide, an antioxidant alkaloid obtained from brown algae *Dictyota coriacea* had a potent effect on regulation of anti‐inflammatory signaling pathway demonstrating its wide applications.^[^ ^122^ ^]^ Although most of the seaweeds species such as *Ulva*, *Chaetomorpha*, *Enteromorpha*, *Sargassum*, *Padina*, *Gelidiella*, *Gracilaria*, *Acanthophora*, and *Jania* showed positive alkaloid content following methanolic and ethanolic extraction methods.^[^ ^101^ ^]^ Furthermore, among the alkaloid containing species identified by Kumbhar et al.,^[^ ^101^ ^]^ *Gracilaria corticata* and *Acanthophora specifera* contained 9.60–9.07 mg g^−1^ of alkaloid followed by *Chaetomorpha*, *Enteromorpha*, and *Sargassum* species with 5.7–5.4 mg g^−1^ of alkaloid. In another study, it was observed that the green seaweed *Ulva lactuca* and brown seaweed *Stoechospermum marginatum* collected from the Oman Coastal regions contained alkaloids in high percentage with specific antibacterial activity.^[^ ^10^ ^]^ Recently, a study on bioactivity analysis of the red seaweeds *Jania rubens*, *Corallina mediterranea*, and *Pterocladia capillacea* showed that these bioactive rich seaweeds had strong anti‐microbial activity.^[^ ^120^ ^]^

Saponins from various plants that have already been studied include *Yucca schidigera*, *Quillaja saponaria*, *Acacia auriculiformins*, *Sapindus saponaria*, *Sesbania sesban*, and *Medicago sativa*. Patra and Yu^[^ ^131^ ^]^ investigated the effect of saponins from the bark of *Quillaja saponaria* Molina plants at a dose of ≈0.6 g L^−1^ on methanogenesis, rumen fermentation, and microbial community and found that the saponin alone decreased the protozoal community and supported CH_4_ mitigation. Belanche et al. (2015),^[^ ^21^ ^]^ reported the brown seaweeds *A. nodosum* and *Laminaria digitata* had effects on rumen fermentation and displayed anti‐protozoal activity thought to be due to the saponins content of the seaweeds. *A. nodosum* produced a 23% decrease in protozoal activity in ruminal fluids obtained from four barren, rumen‐cannulated Holstein‐Friesian cows and simultaneously decreased CH_4_ emissions by ≈15% upon addition as a feed additive. Contrastingly, *L. digitat*a did not show any effect on protozoal activity but modified the rumen fermentation pathway promoting a linear increase in propionate thereby decreasing CH_4_ emissions (Belanche et al., 2015).^[^ ^21^ ^]^ In both studies, the effect of seaweed saponins cannot be ruled out but requires further refinement (Belanche et al., 2015).^[^ ^21^ ^]^ In this study, although the dry matter intake was kept constant, the milk production or composition was not studied. A study conducted by Kumbhar et al.^[^ ^101^ ^]^ reported that various seaweed species including *Ulva*, *Chaetomorpha*, *Enteromorpha*, *Gelidiella*, *Gracilaria*, *Acanthophora*, and *Jania* had a high level of saponinsthat could be extracted using methanol and ethanol. In another study, Anjali et al.^[^ ^10^ ^]^ characterized *Ulva lactuca* (green) and *Stoechospermum marginatum* (brown) seaweeds and showed that these seaweeds were rich in saponins with profound antibacterial activity. Few other green (*Ulva reticulata*) and brown (*Sargassum wightii*) seaweeds contained saponins with promising antibacterial activity.^[^ ^84^ ^]^ Abbott et al.^[^ ^1^ ^]^ summarized the various saponin content of different seaweed species and reported that the saponin content usually ranged between 13 and 17% with respect to dry weight of the alga. Documented evidences also indicated that saponins isolated from red seaweed with an overall diet inclusion rate of 3.6% resulted in decreased protozoal populations which inhibited methanogenesis in sheep.^[^ ^136^ ^]^

## 10 Carotenoids

Seaweeds are a rich source of carotenoid compounds but their content and variety depend on the type of seaweed. Green seaweeds mainly contain chlorophyll but other carotenoids include β‐carotene, lutein, violaxanthin, and the red seaweeds predominantly contain phycoerythrin and phycocyanin along with other carotenoids such as α and β‐carotene, lutein, zeaxanthin and a trace amount of chlorophyll.^[^ ^122^ ^]^ Likewise, brown seaweeds predominantly contain fucoxanthin and other small quantities of β‐carotene, violaxanthin, diatoxanthin, and traces of chlorophyll.^[^ ^122^ ^]^ Numerous studies have already been conducted on seaweed carotenoids showing anti‐inflammatory responses but these studies lack data on antimicrobial activity affecting rumen methanogenesis.^[^ ^122^ ^]^ A study conducted on in vitro ruminal fermentation characteristics with extracts from the brown seaweed *Ecklonia stolonifera* showed that the level of reduction in CH_4_ emissions was increased but upon validation using PCR technique it was observed that the extracts helped in lowering *Fibrobacter succinogenes* populations.^[^ ^104^ ^]^ However, the exact mechanism of action and plausible cause for increase in CH_4_ emissions were not explained. Mohy El‐Din and El‐Ahwany et al.^[^ ^120^ ^]^ reported the highest carotenoid content in the red alga *Pterocladia capillacea* and the lowest content in *Corallina mediterranea* with antioxidant activities. Costa et al.^[^ ^45^ ^]^ reported the carotenoid concentrations of brown, green, and red seaweeds including *Ascophyllum* sp. (brown) ≈1.5–737 mg kg^−1^, *Laminaria* sp. (brown) ≈25.7 mg kg^−1^, *Undaria pinnatifida* (brown) ≈54.4 mg kg^−1^, *Ulva* sp. (green) ≈169–2550 mg kg^−1^, and *Porphyra* sp. (red) ≈72.7–1630 mg kg^−1^. The study also supported the idea of using seaweeds as a feed supplement as the investigation showed that carotenoids were contributing to enhanced meat shelf‐life and meat quality upon inclusion at 2% of DM.

## 11 Application and Effect of Seaweed Bioactives in Studies Concerning Reduction of CH_4_ Emissions from Livestock

Seaweeds can be fed as a source of nutrients and more recently are examined for their potential to reduce emissions from cattle, sheep, and dairy cows. Campbell et al.^[^ ^35^ ^]^ looked at the ensiling effect of seaweeds *Fucus vesiculosus* and *Saccharina latissimi* and subsequent addition as ruminant feed ingredients for a period of 90 days and the results showed no loss in nutrient content of the seaweeds. During the silage process a homo‐fermentative pattern was observed that resulted in lactic acid production supporting lowering of enteric CH_4_ emissions.^[^ ^35^ ^]^ Previous studies related to bioactive compounds present in *A. nodosum* on animal performance showed that the bioactives increased degradability of proteins, enhanced forage digestibility, and linearly reduced in vitro fermentation, while lowering the pathogenic load and increased food safety.^[^ ^115^ ^]^ It was reported that other than *A. nodosum*, *Laminaria* and *Saccharina* were also fed to sheep during the summer at low tide. A 90% diet inclusion rate of the total DMI resulted in a high seaweed consumption rate but animals suffered from mineral overload.^[^ ^115^ ^]^ Other seaweeds that were used as feed ingredients were *M. pyrifera* (≈30% diet inclusion rate in goats); *Sargassum* sp. (≈30% diet inclusion rate in sheep and goats); and *U. lactuca* (≈20% diet inclusion rate in lambs).^[^ ^115^ ^]^ Brooke et al.^[^ ^33^ ^]^ showed that the brown seaweed *Zonaria farlowii* which is native to Southern California when fed at a dose of 5% DMI resulted in 11% reduction in CH_4_ production during in vitro rumen fermentation in Holstein cow. In this study, the Br_3_CH_3_ concentration was found to be very low (≈35 µg g^−1^ dry weight) and neutral detergent fiber (41.7 µg g^−1^ dry weight) content and iron (1765 µg g^−1^ dry weight) and non‐fiber carbohydrate (23.7 µg g^−1^ dry weight) were comparatively higher.^[^ ^33^ ^]^ However, the reduction in CH_4_ showed no obvious impact on total gas and CO_2_ production. Moreover, the released CO_2_ was used as a highly sensitive proxy for detecting changes in the overall metabolic carbon respiration and growth of a microbial community within the microenvironment.^[^ ^33^ ^]^ In a different study, the application of non‐conventional roughages to reduce enteric CH_4_ emissions revealed that lower fiber content feed blocks along with higher levels of flavonoids, phenols, saponins, and antioxidant activity were successfully able to reduce enteric CH_4_ emissions by 26.8–49.3% in sheep.^[^ ^22^ ^]^ Similarly, Dhanasekaran et al. (2020),^[^ ^184^ ^]^ in their study also recommended that the plant secondary metabolites and bioactive compounds containing tannins, essential oils, and saponins hugely impacted the enteric CH_4_ emissions in a natural, positive way. However, due to limited data on these bioactives exact mechanisms of action were unclear. Fagundes et al.^[^ ^52^ ^]^ demonstrated the effect of condensed tannin rich forages on lowering enteric CH_4_ emissions in beef cattle and showed that condensed tannins were able to alter the microbial consortium.

The mechanisms of action regarding methane reduction vary—it could be antibacterial activity, it could be antioxidant effects or it could be a shift in the type of bacteria present in the rumen that causes the observed methane reductions.

Finally, there is a reason why methane is produced in the rumen – to mitigate the excess of H_2_ produced during carbohydrate fermentation. Eliminating methane‐producing bacteria may impact rumen buffering and microbial protein synthesis which are extremely important for ruminant health. This often affects fiber digestion and intake. In addition, some bacteria may stop synthesizing methane (shifting to another pathway), and new bacteria might grow instead. It is important to get the balance right between reducing methane and maintaining animal health.

## 12 Seaweed Production and Techno‐Economic Feasibility Analysis

### 12.1 Production Volumes and Location

Seaweed farming has become a robust form of aquaculture that has increased in terms of production exponentially from 7 million tonnes to 24 million tonnes of biomass productivity between 1997 and 2012.^[^ ^53^, ^68^ ^]^ Rebours et al.^[^ ^140^ ^]^ reported that seaweed for human consumption alone constituted about 83% of the total production rate and the remainder was used for animal feed additives, medicinal purposes, and biotechnological applications. As of 2016, more than 30.2 million tonnes (wet weight) of seaweeds were produced globally, of which 98% came from aquaculture.^[^ ^8^, ^54^ ^]^ It is forecasted that the market value of 30.2 million tonnes of seaweed would be US$30.2 billion by 2025.^[^ ^54^ ^]^ Aquaculture contributed to 96% (99.05% by quantity and 99.36% by value) of global seaweed production with Asian countries dominating throughout followed by Latin America and Europe, of which 40.7% was from the tropical seaweed species such as *Kappaphycus* sp. And *Eucheuma* sp.^[^ ^8^, ^51^ ^]^ Such a jump in production volume indicated that wild harvest alone contributed less significantly, perhaps, the majority of the harvest was from aquacultural fresh produce. In terms of seaweed cultivation, the scale of cultivation in Europe is much less than that found in Asia, particularly, China and countries including Indonesia, Korea, the Philippines, and Japan. In Europe, the majority of seaweeds are harvested from the wild areas but countries including Norway, France, and others are focusing on aquaculture in recent times.^[^ ^56^ ^]^ The volume and location of seaweed cultivation across the globe are represented in **Table** **2**.

**Table 2:** Volume and location of seaweed cultivation

Species	Seaweed type	Location	Volume and yield	Ref.
Latin America, America, and Canada				
Carrageenophytes: *Sarcothalia crispata*, *Mazzaella laminarioides*, *Gigartina skottsbergii*, *Chondracanthus chamissoi* Agarophytes: *Gracilaria chilensis*, *Gelidium lingulatum*, *Callophyllis variegate*, and *Chondrus canaliculatus*	Red	Chile	Aquaculture vol: 14 469 tonnes (wet weight) Yield: US$21 848 000 Harvest vol: 417 965 tonnes	[70]
*Gracilaria lamaneiformis*, *Porphyra columbina*	Brown	Peru	Harvest vol: 5801 tonnes	[140]
*Gelidium robustum*, *Chondracanthus canaliculatus*, *Gracilariopsis lemaneiformis*	Red	Mexico	Harvest vol: 5721 tonnes	[140]
*Gracilaria* spp. and *Hypnea musciformis*	Sea lettuce	Brazil	Harvest vol: 730 tonnes Yield: US$3.50 per dry kg of biomass	[70, 140]
*Saccharina latissima*	Brown and kelp	USA	Harvest vol: 6500 metric tonnes Yield: US$ 1 million	[94]
*Ascophyllum nodosum*	Brown	Canada	Harvest vol: 40 000 tonnes Yield: US$ 40 million	[140]
Europe				
*Laminaria hyperborean*, *Ascophyllum nodosum*	Brown	Norway	Harvest vol: 150 000 tonnes Yield: 23 euros per tonne (wet weight)	[116a]
*Gracillaria* sp. and *Laminaria* sp.	Brown and sea lettuce	Denmark	Harvest vol: 40 000 tonnes Yield: US$564 per metric tonne	[161]
*Saccharina latissima*	Kelp	Sweden	Harvest vol: 8 tonnes per km long line per year (wet weight)	[68]
*Ascophyllum nodosum*, *Feamainn bhuí*, and *Laminaria hyperborea*	Red, rockweed, and kelp	Ireland	Harvest vol: 29 500 tonnes	[121]
*Gelidium* sp.	Red	Portugal	Harvest vol: 2328 tonnes	[121]
*Ascophyllum nodosum*, *Laminaria hyperborean*, *Saccharina latissima*	Rockweed, kelp, Tangle	Iceland	Harvest vol: 17 985 tonnes	[59]
*Laminaria hyperborean*, etc.	Kelp and Sea lettuce	Scotland	Harvest vol: 33 000 tonnes per year	[26]
*Ulva rigida*, etc.	Sea lettuce	France	Harvest vol: 70 000 tonnes per year	[26]
*Gelidium* sp., *Laminaria* sp., etc.	Brown and green	Spain	Harvest vol: 3493 tonnes	[121]
Asia				
*Pyropia* sp., *Undaria* sp., *Saccharina* sp.	Red and brown	Korea	Harvest vol: 1 761 526 tonnes Yield: US$ 525 million	[78]
*Pyropia* sp., *Undaria* sp., *Saccharina*, *Monostroma*, *Ulva* sp., and *Cladosiphon* sp.	Red, brown, and sea lettuce	Japan	Harvest vol: 343 300 tonnes wet weight per year Yield: US$ 1 billion	[170]
*Pyropia* sp., *Undaria* sp., *Saccharina* sp., *Gracilaria* sp.	Red and brown	China	Harvest vol: 12 819 485 tonnes wet weight per year	[150]
*Eucheumatoids* (*Kappaphycus* and *Eucheuma* sp.), *Gracilaria* sp.	Red and brown	Indonesia	Harvest vol: 8 971 463 tonnes wet weight per year	[150]
*Eucheumatoids* (*Kappaphycus* and *Eucheuma* sp.)	Red and brown	Malaysia	Harvest vol: 245 332 tonnes wet weight per year	[150]
*Eucheumatoids* (*Kappaphycus* and *Eucheuma* sp.)	Red and brown	Philippines	Harvest vol: 1 549 576 tonnes wet weight per year	[150]
Australasia				
*Ecklonia radiate*, *Undaria* sp., *Lessonia corrugata*	Brown and Kelp	Australia	Newly developed industry	[158]
	Africa			
*Ecklonia maxima* and *Laminaria pallida*	Brown	South Africa	Newly developed industry	[145]

According to FAOSTAT,^[^ ^55^ ^]^ four temperate and five tropical countries were able to produce around 99.3% of farmed seaweeds, wherein wild harvest was limited to ≈1 million tonnes per year with an increase of 4% in the following year. In the year 2012, total farmed and wild‐harvest was ≈25 million tonnes representing only 0.3% of the total plant‐food produced by the agriculture sector.^[^ ^138^ ^]^ For instance, biomass yield of kelp (rate: 39.7 g dry weight m^−2^ day^−1^; yields: 600 t ha^−1^ year^−1^) was observed to increase by 20% from what was already reported 40 years ago.^[^ ^138^ ^]^ Among the top seaweed cultivars *Eucheuma* spp. (mainly *E. denticulatum*) and *K. alvarezii* with a yield of 8.26 Million tonnes (wet weight) equivalent to 33.3% of world production was the most popular seaweed followed by *Saccharina (Laminaria) japonica* (yield: 5.76 million tonnes; world production %: 23.2); *Gracilaria* spp. (yield: 2.83 million tonnes; world production %: 11.4); *Undaria pinnatifida* (yield: 2.14 million tonnes; world production %: 8.6); and *Pyropia (Porphyra)* spp. (yield: 1.81 million tonnes; world production %: 7.3).^[^ ^55^ ^]^ Subsequently, in 2016 high volumes of seaweed biomass (16 218 406 tonnes equivalent to 53.8% of global seaweed production) were produced by the 3 Asian countries that is, Korea, Japan, and China sharing similar seaweed flora.^[^ ^79^ ^]^ Further, around 47 seaweed cultivars were certified for commercial use with *Saccharina japonica*, *Pyropia* spp., *Undaria* spp., *Cladosiphon okamurarus*, and *Nemacystus decipiens* as the key commercial species in these 3 countries.^[^ ^79^ ^]^ A steady growth in seaweed farming was observed in Indonesia, wherein the industry grew from 0.2 million tonnes in 2003 to 6.5 million tonnes in 2012.^[^ ^138^ ^]^ Such increase in production volumes demonstrates a few suitable factors that allowed the industry to expand, including i) suitable environmental cultivation; ii) it is expected that shifting toward seaweed usage can increase the probability of other value added refined chemical production generating revenues. Additionally, from the environmental perspective, Neori^[^ ^125^ ^]^ pointed out that the marine phytoplankton was able to fix around 50 picogram of carbon equivalent to 10^15^ g per year, and therefore, nurturing such farming activity was believed to lessen the pressure generated by the animal industry in order to maintain a balancing act (**Table** **3**).

**Table 3:** List of companies currently registered for seaweed‐based bioproduct commercialization

Company name	Headquarter	Seaweed Species	Product	Revenue
Acadian Seaplants Limited (ASL)	Nova Scotia, Canada	*Ascophyllum nodosum*	Sea vegetables; crop stimulant; nutritional products; and animal feed supplement	US$30 million
Cargill Incorporated	Minnesota, United States	*Gigartina*, *Chondrus*, *Iridaea*, and *Eucheuma*	Commercially available carrageenans	US$114.695 billion
DuPont de Nemours, Inc.	Delaware, United States	–	Carrageenan products tailored to targeted applications	US$21.57 billion
Irish seaweeds	Northern Ireland	*Palmaria palmata*, *Saccharina latissimi‐Laminaria saccharina*, *Ulva lactuca*, *Himanthalia elongata*, and *Alaria esculenta*	Sea vegetables	€30 million per annum
Beijing Leili Marine Bioindustry Inc. (Leili Group)	Beijing, China	Brown seaweed	Plant nutrients, fertilizers, and supplements	US$50 million
Mara seaweed	Edinburgh, Scotland	Brown seaweed, Kombu	Human nutrition	US$48.40 million
Qingdao Gather Great Ocean Algae Industry Group (GGOG)	Qingdao, China	–	Carrageenan, feed, and nutrition	US$1.2 billion
Algaia	France	–	Seaweed extracts for food, cosmetics, dietary supplements, and agriculture	US$17.83 million
Marinova Pty Ltd	Australia	*Sargassum* sp.	Organic fucoidan, algal polysaccharides	US$5.18 million
Glycomar	UK		Immunomodulatory properties	US$31.0 million
AfriKelp	Cape Town, South Africa	*Ecklonia maxima*	Liquid seaweed extracts for agriculture	US$1.67 million

The United States of America is at a very infancy stage in seaweed aquaculture in comparison to Asia, followed by Europe, however, in the year 2018 the US Department of Energy supported 18 innovative projects for developing offshore seaweed aquaculture technology of worth UD$22 million.^[^ ^94^ ^]^ The program is enunciated as MARINER and is considered as one of the largest investments in the world for offshore seaweed aquaculture developing critical tools, whilst enhancing the nascent macroalgal industry across USA.^[^ ^94^ ^]^ Perhaps, it was believed that through the implementation of this project best opportunities for seaweed aquaculture shall be acquired as follows: i) exploration of sufficient space for aquaculture without conflicting recreational or other fishing activities; and ii) route to permit seaweed aquaculture legally. Biggest motivation to encourage seaweed aquaculture was in maintaining natural balance in the ecosystem as the seaweeds are widely known to remove excess nutrients like carbon and nitrogen from varied habitats whilst reducing ocean acidification.^[^ ^160^ ^]^ The idea of removing excess nutrients by cultivating seaweeds is known as “nutrient bio‐extraction” and the technology has been certified by US EPA as the best management practice with environmental and social benefit.^[^ ^160^ ^]^ In spite of the positive impact of seaweed aquaculture limited growth in the industry can be observed for US, thus more awareness about positive environmental effect and gaining public acceptance throughout the continent must be emphasized. Moreover, certain questions need to be addressed in the market for seaweed‐based ruminant mitigation domain and they are as follows: i) Is there a seaweed market for ruminants?; ii) Can we feed those to ruminants commercially now? iii) If not, what are the main things keeping that from happening?; iv) What are the main challenges for the industry to commercially use seaweed for ruminants in the US and worldwide? As of now, the market for seaweed‐based ruminant feed has started mainly using *Asparagopsis* in some countries but is at the R&D trial stage conducted in conjunction with the dairy industry. A detailed report from these trial runs needs to be thoroughly studied whilst future opportunities need to be evaluated carefully before starting its application in the US and worldwide.

### 12.2 Companies and Process Types and Costs

Of the total global seaweed production 75 to 85% are used directly for human consumption and the rest are used as thickening agents.^[^ ^34^ ^]^ According to a Nayar and Bott^[^ ^124^ ^]^ report, carrageenan had the highest market value of US$ 527 million (raw material value: US$1400 per tonne; final product value: US$10 500 per tonne) followed by alginate with market value of US$318 million (raw material value: US$950 per tonne; final product value: US$12 000 per tonne); agar of market value US$173 million (raw material value: US$1200 per tonne; final product value: US$18 000 per tonne); soil additives of market value US$30 million (raw material value: US$18 per tonne; final product value: US$20 per tonne); fertilizer of market value US$10 million (raw material value: US$500 per tonne; final product value: US$5000 per tonne); and seaweed meal of market value US$10 million (raw material value: US$100 per tonne; final product value: US$500 per tonne). One of the persistent questions to discuss are as follows: i) seaweed production cost with respect to product value, and ii) production and processing cost envisioned in comparison to the existing production process. There are a number of reports suggesting lower production cost ≈US$155 per tonne dry matter to high production cost ≈US$16 630 per tonne dry matter from 1998 to 2011.^[^ ^34^ ^]^ According to a recent finding it was evaluated that seaweed production cost in the North Sea, Atlantic Ocean region resulted in much higher production cost ≈ €1850 per tonne dry matter, however, following an offshore long‐line seaweed cultivation system in exposed deep water locations (>50 m deep) the cost of seeding material and cost of deployment were significantly reduced.^[^ ^13^ ^]^ For instance, the total cost per kg dry matter was decreased from €3673 to €927 per tonne dry matter upon reducing re‐seeding whilst cultivating *Saccharina latissimi*.^[^ ^13^ ^]^ In another study, combined effect of offshore wind farm application along with offshore aquaculture resulted in 10% decrease in production cost.^[^ ^142^ ^]^

In Europe, emerging seaweed industries are driven by innovation in production and growth mainly for human consumption purposes and some of the highly visible examples are seaweed pasta, cheese, and burgers.^[^ ^34^ ^]^ Particularly, seaweed burgers were introduced in the year 2012 by a Dutch seaweed farm called “Zeewar” and became very popular in Amsterdam since 2017.^[^ ^34^ ^]^ In this context a company named Olijck brought several seaweed‐based products such as seaweed ravioli and tagliatelle since 2015. Likewise, Seamore, an Amsterdam based company started producing pasta and bacon made with seaweed/wrapped in seaweed.^[^ ^34^ ^]^ Apart from all these initiatives, seaweeds for bioactive compound production and application for enteric ruminant fermentation are scanty. As of now, the global market for seaweed bioactive is oriented toward food additive application only unlike other applications such as methane gas reduction in livestock. Interestingly, seaweeds containing huge pool of chemical precursors and macro‐chemicals could be potentially used for reducing enteric methane, though the products for these markets are either at subject of study or commercially available at a very small scale. Perhaps, specialized seaweed industries producing particular seaweed type for enteric methane reduction are very limited. A few examples, a Swedish start‐up company “Volta Greentech” just received US$ 500K funding to expand their small scale to pilot scale production of red seaweed “*Asparagopsis*” for enteric methane reduction. Likewise, an Australia‐New Zealand based aquaculture start‐up company “CH_4_ Global” received US$3 million funding leveraging enteric methane reduction by *Asparagopsis armata*. However, current research indicates that farming seaweeds rich in bromoform is more dangerous to the environment including humans as the compound is carcinogenic and also accelerates ozone layer depletion.^[^ ^1^ ^]^ Therefore, seaweed farming focusing on non‐bromoform containing species capable of producing natural bioactive compounds having positive effect on enteric methane reduction could be potentially valuable.

## 13 Current Status

Globally, demand for seaweed for use as animal feed has increased in recent years. It is a recognized source of vitamins and minerals and is often found as an ingredient in dietary supplements for animals, especially ruminants in the form of licks or salt additives. The economic value of the enteric methane mitigation technologies for the Australian red meat industry up to 2030 was thoroughly reviewed by Davison et al.^[^ ^47^ ^]^ While conducting the economic evaluation benefits of each technology used were calculated wherein financial gains from improvements to productivity and carbon credits were considered.^[^ ^47^ ^]^ This study also indicated that *Asparagopsis*‐based mitigation techniques are expected to result in net positive value benefiting the industry around $2600 from 2020–2030 in Australia. Such values were calculated by considering these assumptions as follows: *Asparagopsis* production cost ≈US$7.50 kg^−1^ at the rate of 0.2% of feed intake for only 60 days per year.^[^ ^47^ ^]^ As of March 2020, the average price for each Australian carbon credit and the benefits from emission reduction payments were found to be 16.14 AUD/t CO_2_‐e. In addition, the study showed that after *Asparagopsis*‐based mitigation technique, ruminant microbial manipulation technique also holds potential in benefiting both the industry ≈$1000 M. However, study by Davison et al.^[^ ^47^ ^]^ could show positive directive for red meat industry but lacks a thorough analysis of breakeven points for such calculations. As, discounted %, raw material, labor cost, downstream processing cost, product recovery cost, and supply chain cost were not clearly described. On the other hand Sadhukhan et al.^[^ ^146^ ^]^ while investigating the life cycle assessment of seaweed biorefinery, reported that the production cost was estimated to be $2010 t^−1^ which was significantly lower than their actual product market price. Moreover, a large number of seaweed economics can be found from 1998 to 2011, wherein some studies indicated the production cost as low as US$ 155 per tonne dry matter (DM) or as high as US$16 630 per tonne dry matter.^[^ ^34^ ^]^ In recent times, updated reports on economic feasibility of brown seaweed production based on economic modeling revealed that the seaweed production cost in the North Sea region would be ≈€1850 per tonne DM.^[^ ^34^ ^]^ While economic models for seaweed farming are well documented, however, economic models on seaweed bioactives for enteric methane mitigation are particularly limited. For instance, Sadhukhan et al.^[^ ^146^ ^]^ reported a clear picture of seaweed protein, sugar‐based chemicals, and inorganics showing highest to lowest climate change impact saving 12, 3, and 1 kg CO_2_ equivalent to per kg of the product but did not showcase about the economics of bioactive usage in livestock. After going through rigorous searches, a few articles are found on *Asparagopsis‐*based enteric methane mitigation. Although *Asparagopsis*‐based techniques seem to be rewarding at this stage in reality the techniques are stemmed around several other negative factors such as environmental and health impacts caused due to bromoform release. Also, in order to materialize the technique several other economic, environmental, and livestock and human health studies need to be conducted.

## 14 Conclusion

The potential of seaweed to mitigate methane is real and studies with red seaweeds have found reductions in methane produced from ruminants in the region of 60–90% in the case of seaweed like *Asparagopsis taxiformis* where the active ingredient is bromoform. Other studies with brown and green seaweeds have observed reductions in methane production of between 20 and 45% in vitro and 10% in vivo. The benefits of feeding seaweeds to ruminants are seaweed species and animal species dependent. In some instances, positive effects were observed in milk production and performance when selected seaweeds were fed to ruminants while other studies note reductions in performance traits. A balance between reducing methane and maintaining animal health and food quality is necessary. Seaweeds are a source of essential amino acids and minerals however and offer huge potential for use as feeds for animal health maintenance once formulations and doses are correctly prepared and administered. A negative aspect of seaweed use for animal feed currently is the cost associated with wild harvest and indeed aquaculture production and improvements must be made here if seaweed ingredients are to be used as a solution to methane production from ruminants for continued production of animal/ruminant sourced proteins.

## Conflict of Interest

The authors declare no conflict of interest.
